# Transcriptome Analysis of MSC and MSC-Derived Osteoblasts on Resomer® LT706 and PCL: Impact of Biomaterial Substrate on Osteogenic Differentiation

**DOI:** 10.1371/journal.pone.0023195

**Published:** 2011-09-14

**Authors:** Sabine Neuss, Bernd Denecke, Lin Gan, Qiong Lin, Manfred Bovi, Christian Apel, Michael Wöltje, Anandhan Dhanasingh, Jochen Salber, Ruth Knüchel, Martin Zenke

**Affiliations:** 1 Institute of Pathology, Rheinisch-Westfälische Technische Hochschule Aachen University, Aachen, Germany; 2 Biointerface Group, Institute for Biomedical Engineering, Rheinisch-Westfälische Technische Hochschule Aachen University, Aachen, Germany; 3 Helmholtz Institute for Biomedical Engineering, Rheinisch-Westfälische Technische Hochschule Aachen University, Aachen, Germany; 4 Interdisciplinary Centre for Clinical Research, IZKF Aachen, Rheinisch-Westfälische Technische Hochschule Aachen University, Aachen, Germany; 5 Department of Cell Biology, Institute for Biomedical Engineering, Rheinisch-Westfälische Technische Hochschule Aachen University, Aachen, Germany; 6 Electron Microscopic Facility, University Clinics Aachen, Aachen, Germany; 7 Department of Conservative Dentistry, Periodontology and Preventive Dentistry, Rheinisch-Westfälische Technische Hochschule Aachen University, Aachen, Germany; 8 DWI e.V. and Institute of Technical and Macromolecular Chemistry, Rheinisch-Westfälische Technische Hochschule Aachen University, Aachen, Germany; 9 Chirurgische Klinik und Poliklinik, BG Universitätsklinikum Bergmannsheil, Ruhr-Universität Bochum, Bochum, Germany; Centro Cardiologico Monzino, Italy

## Abstract

**Background:**

Mesenchymal stem cells (MSC) represent a particularly attractive cell type for bone tissue engineering because of their *ex vivo* expansion potential and multipotent differentiation capacity. MSC are readily differentiated towards mature osteoblasts with well-established protocols. However, tissue engineering frequently involves three-dimensional scaffolds which (i) allow for cell adhesion in a spatial environment and (ii) meet application-specific criteria, such as stiffness, degradability and biocompatibility.

**Methodology/Principal Findings:**

In the present study, we analysed two synthetic, long-term degradable polymers for their impact on MSC-based bone tissue engineering: PLLA-co-TMC (Resomer® LT706) and poly(ε-caprolactone) (PCL). Both polymers enhance the osteogenic differentiation compared to tissue culture polystyrene (TCPS) as determined by Alizarin red stainings, scanning electron microscopy, PCR and whole genome expression analysis. Resomer® LT706 and PCL differ in their influence on gene expression, with Resomer® LT706 being more potent in supporting osteogenic differentiation of MSC. The major trigger on the osteogenic fate, however, is from osteogenic induction medium.

**Conclusion:**

This study demonstrates an enhanced osteogenic differentiation of MSC on Resomer® LT706 and PCL compared to TCPS. MSC cultured on Resomer® LT706 showed higher numbers of genes involved in skeletal development and bone formation. This identifies Resomer® LT706 as particularly attractive scaffold material for bone tissue engineering.

## Introduction

Human multipotent mesenchymal stem cells (MSC) are multipotent stem cells and represent a particularly attractive source for tissue engineering, because they are readily isolated and expanded and can differentiate into several mature cell types, such as adipocytes, chondrocytes, osteoblasts and myocytes [1], [2 and references therein]. Due to their mesenchymal origin and their osteogenic differentiation capacity, MSC are particularly promising cells for bone replacement. Using a specific culture medium, they can differentiate towards osteoblasts *in vitro* within three weeks [3]. However, a biomaterial scaffold is required for bone tissue engineering (BTE) to allow for immobilisation of cells in a spatial structure. A variety of potential biomaterials exist, which are cytocompatible for MSC, e. g. collagen, fibrin and non-oxide ceramics [4]–[8]. For scaffolds in general, cyto- and biocompatibility are the criteria of upmost importance and they need to meet further specific application-dependant criteria. For BTE, a scaffold should be osteoinductive or at least osteoconductive and has to be long-term degradable to allow for an autologous replacement of the transplanted area. Besides such specific requirements, a fundamental bottleneck must be overcome to use MSC for BTE – the adequate supply of the cells. This problem will become more critical when aiming at engineering of bulk tissue to fill bone defects after injury or elimination of tumors, particularly when autologous bone production is desired. Such goals necessitate the maintainance of large numbers of undifferentiated cells embedded in biocompatible matrices to provide sufficient starting material.

A number of commercially available cell culture materials is accessible, such as standard tissue culture poly(styrene) (TCPS), Primaria™, poly(ethylene terephthalate) (PET) and TC plastics biocoated with Matrigel™ or single extracellular matrix proteins, but given properties of the polymers make them ineligible for tissue engineering. Further, frequently the animal origin of biocoating products disqualify them from use in human clinical applications. Currently, the published literature contains a large number of studies in which stem cells are seeded on one or more readily available materials but frequently such studies lack systematic approaches to determine cell responses.

In our recent work we established a biomaterial test platform to assess the compatibility of stem cells and biomaterials for tissue engineering in a highly standardised and systematic manner. The assessment of stem cell/biomaterial interactions is multifactorial and requires a stringent analysis of parameters, such as material surface and bulk properties, cytotoxicity, cell adhesion, cell morphology, viability, proliferation, necrosis and apoptosis [5]. In our present study, we analysed in more detail the interactions of MSC with two commercially available synthetic and resorbable polymers of our panel of biomaterials, Poly(ε-caprolactone) (PCL) and poly[(L,L-lactide-co-trimethylene carbonate)_7/3_] ( = PLLA-co-TMC = Resomer® LT706).

PCL is a linear, semicrystalline, synthetic aliphatic homopolyester, which has a degradation time of two to three years [9]–[11]. PCL is the most studied degradable polymer of the polyester family [12] and is already approved by the Food and Drug Administration for diverse applications in the human body. Furthermore, PCL is currently under consideration for use in bone tissue engineering [13]–[16].

In contrast, there is only poor knowledge on Resomer® LT706. This linear and semi-crystalline poly-lactide-based polymer is similar in chemistry to Suprathel, which is now used in clinical trials for skin replacement after burns [17], [18]. Further, poly(1,3-trimethylene carbonate) copolymerised with D,L-lactide was shown to be long-term biodegradable and biocompatible for soft tissue engineering [19], but so far, polymers related to Resomer® LT706 were not analysed with respect to their usefulness for bone replacement. The random copolymer PLLA-co-TMC (Resomer® LT706) is characterised by an intermediate-term degradation, slower than poly(glycolic acid) (PGA), poly(D,L-lactide acid) (PDLLA), and poly(glycolic acid-co-trimethylene carbonate) (PGA-co-TMC), but faster than the long-term degradable PCL [20]–[22]. Although, Resomer® LT706 has some advantages regarding its mechanical properties and processing behavior, it is used only in a few studies as potential scaffold for hard and soft tissue engineering [23], [24].

In the present study, we analysed the two polymers in comparison to TCPS for their osteoinductive capacity and thus for their potential use in BTE. Therefore, we seeded MSC on PCL and Resomer® LT706 samples with defined, ultraflat topography to exclude topography-dependant changes in cell adhesion, morphology and proliferation. MSC adhered on both polymers and showed only minor differences in morphology and viability. When subjected to osteogenic induction medium (OIM), MSC differentiated towards the osteoblastic fate. Alizarin red stainings, realtime PCR, scanning electron microscopy (SEM) and energy-dispersive X-ray spectroscopy (EDS) analysis revealed a similar frequency of MSC-derived osteoblasts on the two polymers. However, detailed studies on the molecular level including whole genome expression analysis unravelled differences in the biomaterial-based propensity towards the osteogenic fate of MSC supported by these two polymers.

## Methods

### Polymer synthesis

The polymers were produced as previously described [5] (Neuss *et al.*, 2008a).

### Poly(ε-caprolactone) (PCL)

PCL with a molecular weight of 80.000 g/mol was purchased from Sigma-Aldrich GmbH (Germany). For PCL foils, 3 g of granules were used. These granules were placed on Teflon®-covered metal plates, the temperature was raised to 85°C and maintained for 5 min. A load of 1000 kg was applied for 1 min at 85°C. After cooling down to room temperature, foils were then washed several times with isopropanol (Fluka, Germany), aqueous 0.02 mM Tween 80 (Roth, Germany) and 8 M urea (Roth, Germany) and then rinsed vigorously with demineralised water. PCL samples were then dried in a vacuum oven for 24 h at 40°C. Samples were placed in TCPS wells, stored at 4°C, and protected from light. All steps were carried out under sterile conditions.

### Resomer® LT706

Resomer type poly(L-lactic acid-co-trimethylene carbonate) ( = P(LLA-co-TMC; lactic acid-trimethylene carbonate ratio 70∶30, LT706, 1.2–1.6 dL/g) was purchased from Boehringer Ingelheim Pharma GmbH & Co. KG (Germany). Foils were prepared by melt-pressing technique. Therefore, granules were ground to powder in a cryo-mill and 1.2 g of the powder were placed between Teflon®-covered metal plates. This was followed by a 5 min incubation at 180°C. A load of 1000 kg was applied for 9 min. The P(LLA-co-TMC) foil was allowed to cool down to room temperature, removed from the metal plates and further processed as for as for PCL.

### Coating of biomaterials with radiolabeled fibronectin and vitronectin

100 µl of either fibronectin or vitronectin (100 µg diluted in 100 µl PBS; BD, Heidelberg, Germany) were mixed in a silanised counter vial (3.5 ml volume) with 1 µl Na^125^I solution (0.1 mCi; 3.7 MBq; 45 pmol; Amersham Europe, Freiburg, Germany) and 5 µl Chloramine-T solution (10 µg Chloramine-T trihydrate, solved in 100 µl PBS) using a magnetic stir bar. After 5 min, the incorporation of ^125^I into fibronectin and vitronectin was measured by TCA precipitation. Therefore, a melted glass capillary was immerged first into the iodinising mixture and then into 200 µl of BSA solution (10% (m/m) BSA, 1% (m/m) NaI, 0.01% (m/m) NaN_3_). After vigorously mixing of the solution, an aliquot of 10 µl was admixed with another 200 µl BSA solution. Afterwards, 2 ml ice-cold 10% trichloroacetic acid was added. The precipitated material consisting of BSA and target protein was spinned down, separated from the supernatant and measured using a gamma counter COBRA II Auto-Gamma (Packard, Dreieich, Germany). To separate labelled protein from non-incorporated iodine, the mixture was purified by gel filtration using a Sephadex® G-25 NAP™5 Column (Pharmacia Biotech AB, Uppsala, Sweden). The column was equilibrated with PBS before use. The identified radiolabelled proteins were stored at −20°C. Radiolabeled fibronectin and vitronectin were dissolved in PBS and DMEM with 10% FCS in a concentration of 10 µg/ml.

Radiolabeled protein adsorption study was carried out by incubation of TCPS, Resomer® LT706 and PCL disc surfaces with 2 ml of the different solutions, each with ^125^I-labeled fibronectin or vitronectin (10 µg/ml), respectively. The activity was adjusted to 100.000 cpm/500 µl (1.67 kBq/500 µl; 45.0 pCi/500 µl) using a tracer. To test the resulting activity, samples of 500 µl volume were measured using the gamma counter COBRA II Auto-Gamma during a period of 3 min. After treatment of biomaterial substrates with radiolabelled protein (incubation time 1 h) and subsequent washing, activity was measured using the gamma counter during a period of 5 min.

Significant differences between samples were analysed using student's t-test for 10 independent measurements of each coating.

### Isolation of human mesenchymal stem cells

Human mesenchymal stem cells (MSC) were isolated from femoral head spongiosa of patients with total hip joint endoprosthesis (TEP) after written consent was obtained from the patients. The study was approved by the Ethics Committee of the University Clinics, Aachen, Germany. MSC were characterised as previously described [5]–[8], [25]; Fig. S2. In brief, after rinsing the spongiosa with stem cell medium, spongiosa was removed and the remaining cell suspension was centrifuged for 10 min at 500×g. Thereafter, the cell pellet was resuspended in stem cell medium and cells were seeded in a T75 culture flask and cultured at 37°C in a 21% O_2_ and 5% CO_2_ humidified atmosphere. After 24 hours, non adherent (hematopoietic) cells were removed by medium change. Mesenchymal stem cells were expanded in growth medium (PAN Biotech, Aidenbach, Germany) consisting of 60% DMEM low glucose, 40% MCDB-201, 2% FCS, 1×ITS-plus (insulin-transferrin-selenic acid+BSA-linoleic acid), 1 nM dexamethasone, 100 µM ascorbic-acid-2-phosphate, and 10 ng/ml EGF. Medium was replenished every 3–4 days. At 80–90% confluency, stem cells were trypsinised with stem cell trypsin (CellSystems, St. Katharinen, Germany) and reseeded in a density of 5,000 cells/cm^2^ for optimal proliferation.

### Differentiation of MSC towards osteoblasts

For osteogenic differentiation, MSC were seeded in a density of 3.1×10^4^ cells/cm^2^ on TCPS and on foils of the two polymers PCL and Resomer® LT706. Twenty-four hours after seeding, the growth medium was replaced with osteogenic induction medium (OIM) consisting of DMEM low glucose (PAA, Coelbe, Germany), 100 nM dexamethasone, 10 mM sodium β-glycerophosphate, 0.05 mM L-ascorbic-acid-2-phosphate (all Sigma, Steinheim, Germany) and 10% FCS (PAN Biotech, Aidenbach, Germany). Medium was changed every 2–3 days. After 21 days of osteogenic differentiation, cells were fixed with 70% ethanol for 1 hour, washed three times with demineralised water and then stained with an Alizarin red solution (40 mM, pH 4.1, Sigma) for 10 minutes. Finally, cells were washed three times with PBS (phosphate-buffered saline).

For quantification, the Alizarin red precipitates were solubilised. Briefly, stained samples were incubated with 800 µl acetic acid (10%) for 30 min. Then, supernatant was transferred into a 1.5 ml tube and boiled for 10 min at 85°C, followed by a 5 min incubation on ice. After centrifugation (15 min, 15,000×g), 500 µl of the supernatant were transferred into another 1.5 ml tube and mixed with 200 µl of 10% ammonium hydroxide. Samples of 150 µl were transferred into a 96 well microtiter plate and optical density was measured at 405 nm using a standard ELISA reader.

P-value to detect statistically relevant differences for the different biomaterials was calculated with student's t-test (n = 3 with two replicates each).

### Scanning electron microscopy (SEM)

MSC/polymer biohybrids were fixed in 3% glutaraldehyde for at least 24 hours, rinsed with sodium phosphate buffer (0.2 M, pH 7.39, MERCK, Darmstadt, Germany) and dehydrated by incubating consecutively in 30%, 50%, 70% and 90% acetone and then three times in 100% acetone for 10 minutes. The biohybrids were critical-point-dried in liquid CO_2_, and then sputter-coated with a 30 nm gold layer. Samples were analysed using an environmental scanning electron microscope (ESEM XL 30 FEG, FEI, PHILIPS, Eindhoven, The Netherlands) in a high vacuum environment.

### Energy-Dispersive X-ray Spectroscopy

Energy dispersive X-ray spectroscopy (EDS) is an analytical technique used for element analysis samples. EDS was performed on the XL 30 FEG scanning electron microscope (FEI, Eindhoven, The Netherlands) using an EDAX Falcon Genesis Spectrum 5.21 energy-dispersive X-ray spectroscopy system with an ultrathin window liquid nitrogen cooled Si(Li) X-ray detector (EDAX Inc. Mahwah, NJ, USA). For the EDS an accelerating voltage of 20 kV was used.

### Reverse Transcriptase Polymerase Chain Reaction (RT-PCR)

Total RNA was isolated using the RNeasy mini Kit according to the manufacturers' instructions (Qiagen, Hilden, Germany). Reverse transcription was done with 1 µg of total RNA using the High Capacity cDNA Reverse Transcription Kit (Applied Biosystems, Darmstadt, Germany). PCR was as follows: denaturation at 95°C for 1 min, annealing at 58°C (osteocalcin), 60°C (alkaline phosphatase) for 1 min, extension at 72°C for 1 min (30 cycles), and a final extension at 72°C for 10 min. PCR products were analysed by electrophoresis using a 2% agarose gel and visualised with ethidium bromide. Primer sequences were used as listed in Table 1.

**Table 1:** Primer sequences for PCR and RT-qPCR.

Gene	Sequence (5′→3′)	Amplicon size
ACTB (ß-Actin)	for TGGCACCACACCTTCTACAATGAGCrev GCACAGCTTCTCCTTAATGTCACGC	400 bp
ALPL (alkaline phosphatase)	for CCTCCTCGGAAGACACTCTGrev AGACTGCGCCTGGTAGTTGT	238 bp
BGLAP (Osteocalcin)	for CCCTCACACTCCTCGCCCTATrev TCAGCCAACTCGTCACAGTCC	246 bp
SFRP4	for GCGCACCAGTCGTAGTAATCCrev TTCTTGGGACTGGCTGGTT	72 bp
PRELP	for CAACAACAATAGCATCGAGAAAATCAACrev AGGTGTGGCACGTTCTCCAG	102 bp
COMP	for CAGGGAGATCACGTTCCTGArev GGCCGGTGCGTACTGAC	77 bp
COL11A2 (Collagen 11)	for GACTATCCCCTCTTCAGAACTGTTAACrev CTTCTATCAAGTGGTTTCGTGGTTT	131 bp
ELN (Elastin)	for CCGCTAAGGCAGCCAAGTATGGArev AGCTCCAACCCCGTAAGTAGGAAT	275 bp
CCL2	for TGTCCCAAAGAAGCTGTGATCTrev GGAATCCTGAACCCACTTCTG	84 bp

### RealTime PCR (RT-qPCR)

RealTime PCR was performed using SybrGreen and Taqman technology. Briefly, 10 µl SybrGreen Master Mix (Applied Biosystems, Darmstadt, Germany) was mixed with 1 µl (10 pg) Primer forward, 1 µl (10 pg) Primer reverse, 6.8 µl water and 1.2 µl (60 ng) template. Then the samples were subjected to the following program: initial denaturation at 95°C for 10 min, followed by 40 cycles of denaturation at 95°C for 15 sec and annealing/extension at 60°C for 1 min. Primer sequences and sizes of amplicons are listed in table 1.

### Whole Genome Expression Analysis

For whole genome expression analysis, MSC were seeded in a density of 3.1×10^4^ cells/cm^2^ on TCPS, Resomer® LT706 and PCL and cultured for 21 days in growth medium (GM) or in osteogenic induction medium (OIM). Cells at day 0 served as control. Total RNA was isolated using the RNeasy micro Kit according to the manufacturers' instructions (Qiagen, Hilden, Germany). The RNA quality was assessed using RNA 6000 NanoChips with the Agilent 2100 Bioanalyzer (Agilent; Santa Clara, CA, USA). Probes for the GeneChip® Human Gene 1.0 ST Arrays (Affymetrix, Santa Clara, CA, USA) were prepared and hybridised to the arrays according to the Affymetrix GeneChip® Whole Transcript (WT) Sense Target labeling Assay Manual. Briefly, for each sample, 300 ng of total RNA was reverse trancribed into cDNA using a random hexamer oligonucleotide tagged with a T7 promoter sequence (5′-GAATTGTAATACGACTCACTATAGGGNNNNNN-3′). After second strand synthesis, double-stranded cDNA was used as template for amplification with T7 RNA polymerase to obtain antisense cRNA. Random hexamers were then used to reverse transcribe the cRNA into single stranded sense strand cDNA. The cDNA was then fragmented by UDG (uracil DNA glycosylase) and APE1 (apurinic/apyrimidic endonuclease 1). Fragment size was checked using the Agilent 2100 Bioanalyzer (fragment size between 50–200 bp). Fragmented sense cDNA was biotin-labelled with TdT (terminal deoxynucleotidyl transferase) and probes were hybridised to the GeneChip® Human Gene 1.0 ST Arrays at 45°C for 16 hours. Hybridised arrays were then washed and stained on Fluidics Station 450 and scanned on a GeneChip® Scanner 3000 7G (both Affymetrix).

The image data were analysed with GCOS (Affymetrix). For statistical analysis data were processed by R software (R Development Core Team, 2005). Gene expression levels were normalised with RMA algorithm [26]. Principal component analysis (PCA) and hierarchical clustering were done on whole genome transcripts by the R Stats Package [27]. Hierarchical clustering was performed using Pearson correlation coefficient and the Average linkage method. RankProd, a non-parametric statistic, was employed for identification of differentially expressed genes [28]. Transcripts with a fold change more than 2 and P-value less than 0.01 between two conditions were considered as being differentially expressed. Gene ontology (GO) and pathway over-representation analysis was done using DAVID bioinformatics resources [29].

## Results

### Resomer® LT706 and PCL enhance MSC differentiation towards osteoblasts

**Figure 1:**
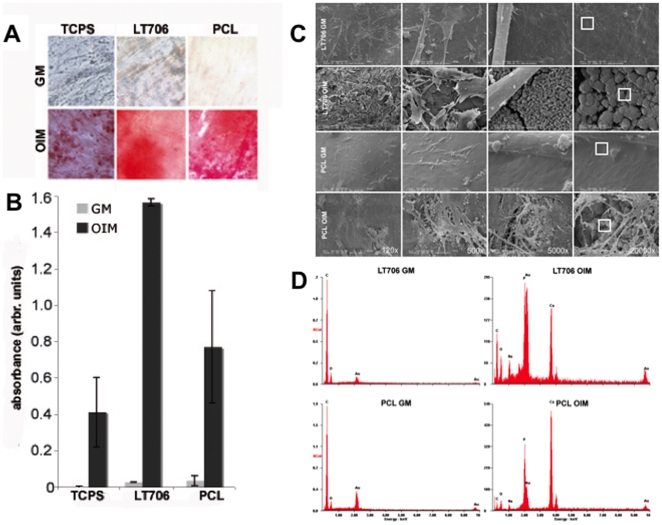
Osteogenic differentiation of MSC on polymers. A) Alizarin red staining of calcium accumulations on MSC, cultured on two polymers and on tissue culture polystyrene (TCPS) for 21 days either in growth medium (GM) or in osteogenic induction medium (OIM). PCL = Poly(ε-caprolactone); LT706 = Resomer® LT706; B) Quantification of Alizarin red staining via dissolving the dye and subsequent absorption measurement (λ = 405 nm); n = 3 with each 2 replicates; p>0.05; C) Electron microscopic view (SEM) of MSC, cultured for 21 days on Resomer® LT706 and PCL either in GM or in OIM. From left to right: higher magnifications of the previous picture (120× up to 20.000×). White boxes in 20.000× magnification images represent the area, which was subjected to EDS analysis (D); D) EDS Analysis of surfaces of Resomer® LT706 and PCL after a 21 day cultivation period with MSC either in GM or in OIM. Prominent peaks of calcium and phosphate were detected in samples cultured in OIM, but not in samples cultured in GM (compare y-axis scale).

In a previous study, we analysed MSC on a grid-based platform in contact to different polymers to determine cytocompatibility of polymers for future MSC-based tissue engineering applications [5]. Now, MSC were cultured for 21 days either in growth medium (GM) or in osteogenic induction medium (OIM) on two of the cytocompatible, synthetic, biodegradable polymers, Resomer® LT706 and PCL, which are potentially osteoinductive. To analyse the osteogenic differentiation, calcium accumulations were visualised by Alizarin red staining (Fig. 1A), followed by dissolving the dye and subsequent quantification of the staining (Fig. 1B). Alizarin red stain residues were not retained by any of the biomaterials (Fig. S3). As shown in Figure 1, polymers did not induce osteogenic differentiation by themselve in GM and OIM was always required to guide MSC into osteogenic fate. However, although OIM was required to initiate osteogenic differentiation of MSC on the two polymers, biomaterial properties influenced calcium accumulations.

**Figure 2:**
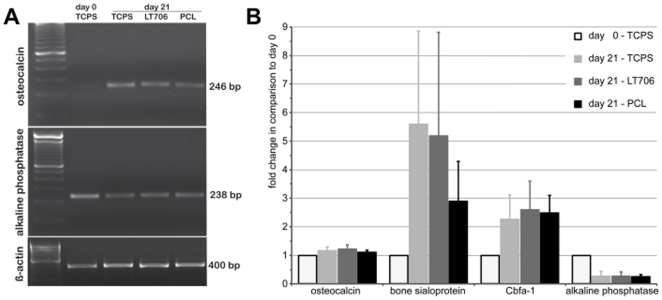
Expression of osteogenic markers on RNA-level. A) Semiquantitative polymerase chain reaction (PCR) of the osteogenic markers osteocalcin (246 bp) and alkaline phosphatase (238 bp) expressed in MSC on TCPS (d0) or cultured for 21 days in osteogenic induction medium (OIM) on TCPS, Resomer® LT706 and PCL. ß-Actin (400 bp), loading control: DNA marker (100 bp ladder) = 600 bp. Results of one representative experiment out of three are shown. B) Microarray-data of three independent experiments (n = 3) for osteocalcin, bone sialoprotein, Cbfa-1 and alkaline phosphatase expressed in MSC on TCPS (d0) or cultured for 21 days in OIM on TCPS, Resomer® LT706 and PCL.

As shown in Figure 1, both polymers resulted in a stronger alizarin red staining than TCPS. Further, the amount of calcium accumulations on Resomer® LT706 was significantly higher, than on TCPS and on PCL. Since the Alizarin red staining is an indirect measure for osteogenic differentiation, we analysed the differentiation in more detail. The ultrastructural analysis depicted in Figure 1C illustrates the morphology of MSC on the two polymers after a three-week culture period in GM or in OIM (Fig. 1C). Our results show that although MSC morphology on the two polymers differed during initial adhesion [5], MSC morphology is identical on both polymers after 21 days of culture and independent of the culture medium (GM vs. OIM). Higher magnifications showed analogous particles densely covering the biomaterial surfaces under differentiation conditions (OIM) on both polymers, which were qualitatively identified as consisting of calcium and phospate by EDS analysis (Fig. 1D). Such calciumphosphate particles are indicative for an advanced osteogenic differentiation of MSC on both polymers, Resomer® LT706 and PCL. These results were confirmed by the expression of osteogenic markers, such as alkaline phosphatase (which is already expressed in unstimulated MSC), osteocalcin, bone sialoprotein and the transcription factor Cbfa-1 after 21 days of cultivation in OIM on the polymers (Fig. 2). By demonstrating (i) positive Alizarin red staining and (ii) expression of standard osteogenic markers on the RNA level, well-accepted standard assays were performed to show osteogenic differentiation of MSC on Resomer® LT706 and PCL. However, we reasoned that conventional standard assays are not sufficient to comprehensively investigate the osteogenic fate of MSC and thus we postulated, that there might be differences in the quality of the MSC-derived osteoblasts on the molecular level. Therefore, we performed whole genome expression analysis to determine biomaterial-related differences in osteogenic differentiation.

### Whole genome expression analysis identifies specific responses of Resomer® LT706

**Figure 3:**
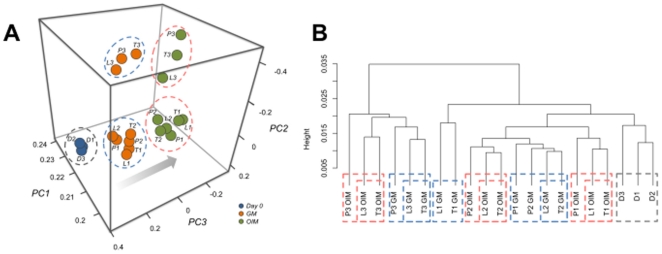
Whole genome expression analysis of MSC, cultured for 21 days on TCPS, Resomer LT706 and PCL in growth medium (GM) or in osteogenic induction medium (OIM). A) Principal component analysis (PCA) demonstrates donor variations, but all donors show the same shift in position upon differentiation. Orange circles = MSC cultured for 21 days in GM; green circles = MSC cultured for 21 days in OIM; blue circles = MSC expanded on TCPS in GM before differentiation experiment; T = TCPS; L = Resomer® LT706; P = PCL; D = Donor at day 0; 1,2,3 = different donors (n = 3); PC1, 2, 3 = Principal Component 1, 2 and 3; B) The dendrogram shows clustering of TCPS and Resomer® LT706, independent of donor and independent of growth (GM) and differentiation (OIM) conditions. T = TCPS; L = Resomer® LT706; P = PCL; D = Donor at day 0; 1,2,3 = different donors (n = 3); red dotted lines = OIM; blue dotted lines = GM.

To determine the molecular events ongoing in MSC differentiation on Resomer® LT706 and PCL, samples were analysed by whole genome expression profiling. First, data were subjected to principal component analysis (PCA, Fig. 3A) to discriminate related samples (similar gene expression) from distant samples (different gene expression). Figure 3A reveals the following information: the closer the samples are, the more related is the gene expression pattern. Thus, PCA demonstrated (i) general changes in gene expression during the 21 day culture period (compare day 0 samples D1–3 cultured in GM, with all other samples), (ii) donor variations, since donor 3 is separated from donors 1 and 2 after 21 days of culture, but (iii) the same shift of all three donors occurred from culture in GM and to culture in OIM (arrow in Fig. 3A). Hierarchical clustering (Fig. 3B) of the same data set also revealed general changes in gene expression over time (samples D1–3, grey dottet line), donor variations as well as the shift between growth and differentiation conditions (blue dottet lines vs. red dottet lines, respectively). Additionally, this analysis also identified clustering of Resomer® LT706 with TCPS, independent of donor and independent of growth (GM) and differentiation (OIM) conditions (Fig. 3B). Thus for each donor, Resomer® LT706 and TCPS consistently clustered together when cultured in GM or in OIM (e.g. L3 OIM/T3 OIM or L2 GM/T2 GM). In most cases, these neighboring clusters were adjacent to the corresponding donor in the respective medium on PCL (e.g. P3 GM is next to the L3 GM/T3 GM pair). Although the Alizarin red staining was much stronger on Resomer® LT706 than on TCPS (Fig. 1A, B), the impact of Resomer® LT706 on MSC gene expression seemed to be comparable to TCPS, both after culture in GM and OIM. In contrast, PCL resulted in a different gene expression profile compared to Resomer® LT706 and TCPS, but in most cases, PCL samples were in direct proximity to TCPS and Resomer® LT706 in the respective culture conditions (blue and red dottet lines; Fig. 3B).

The results shown in Figure 3 indicate donor variations, as expected for primary cells, but a similar influence on gene expression by TCPS and Resomer® LT706, which was independent of culture medium. Hence, we show that culture medium has a stronger influence on MSC differentiation than the biomaterial substrates. Alizarin red staining was stronger for PCL and Resomer® LT706 compared to TCPS (Fig. 1A, B), yet PCL affected more genes within the same culture conditions than Resomer® LT706 when compared to cells on TCPS. This analysis describes the overall impact on the gene expression profile and the identity of differentially expressed genes is discussed below.

### Biomaterial substrate and culture medium impact on gene expression of MSC and MSC-derived osteoblasts

Resomer® LT706, PCL and TCPS are semi-crystalline polymers with a characteristic, textured structure and ultraflat topography. Thus, the biomaterial surface physicochemistry could be responsible for the specific changes in gene expression rather than the biomaterial topography. Depending on the surface physicochemistry, proteins adsorb to the biomaterial, yet the underlaying mechanisms are still not fully understood. In this context we emphasize that cells do not adhere directly to biomaterial surfaces but rather adhere to a protein layer, which promptly adsorbes to biomaterials, when exposed to cell culture medium. Within the two cell culture media (GM and OIM) the composition of proteins is quite different, which presumably results in different protein layers on the biomaterial surfaces when cultured either in GM or in OIM (2% FCS vs. 10% FCS, respectively). Further, the amount of adsorbed proteins and the orientation of active groups of the adsorbed proteins could differ depending on the biomaterial physicochemistry. Accordingly, this leads to different cell behaviours on the very same biomaterial, when cultured in different media. However, within the same culture medium, different biomaterials can influence cells in different ways.

**Figure 4:**
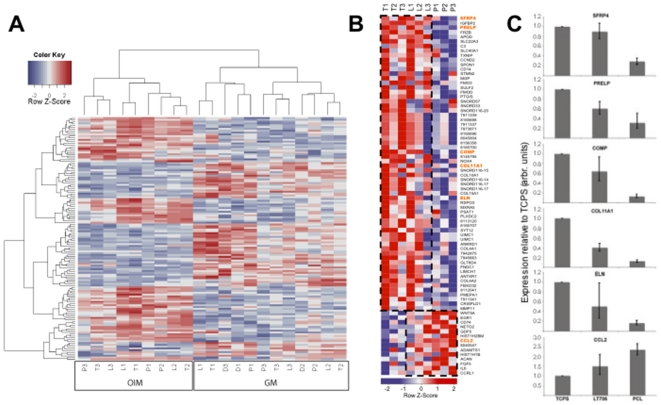
Impact of the biomaterials and culture media on MSC at growth and differentiation state. A) Heatmap representation of medium-dependant effects on MSC, cultured for 21 days on TCPS, Resomer® LT706 and PCL, either in growth medium (GM) or in differentiation medium (OIM). T = TCPS; L = Resomer® LT706; P = PCL; D = donor at day 0; 1,2,3 = different donors (n = 3); B) Heatmap representation of biomaterial impact on differentiation state (MSC cultured in OIM after 21 days). Genes boxed by a discontinuous line are similarly expressed in TCPS and Resomer® LT706 (upper box) or in Resomer® LT706 and PCL (lower box). Genes highlighted in orange were analysed by RT-qPCR. T = TCPS; L = Resomer® LT706; P = PCL; 1,2,3 = different donors; C) RT-qPCR for SFRP4, PRELP, COMP, COL11A1, ELN and CCL2 to confirm gene array results of Figure 4C. Expression of genes was normalised to the reference gene ß-actin. TCPS was used as calibrator. n = 3.

The heatmap of expression data shown in Figure 4A demonstates the impact of biomaterial substrate and culture medium on gene expression of MSC and MSC-derived osteoblasts. We detect two main gene expression pattern: one is representative for cells cultured in GM (Fig. 4A, right) and one for cells cultured in OIM (Fig. 4A, left), regardless of the biomaterial substrate meaning that the impact of the growth medium is higher than the impact of the biomaterials. However, within the two groups, MSC cultured on Resomer® LT706 and TCPS cluster together, while PCL form a separate branch, which is in line with the results depicted in Figure 3. Again, these results demonstrate the influence of culture medium on MSC, which is more prominent than the influence of biomaterial substrates.

As shown by hierarchical clustering (Fig. 3 and Fig. 4A), MSC on Resomer® LT706 and TCPS show a similar gene expression pattern and this translates into a small number of differentially expressed genes. As described above, MSC do not attach directly to the biomaterial surface, but to the protein layer, which covers the biomaterials after exposure to the cell culture medium. As shown in Figure S1, fibronectin (FN) and vitronectin (VN), two main components of serum involved in cell adhesion, are adsorbed to TCPS, Resomer® LT706 and PCL. While the amount of adsorbed FN is very similar for TCPS and PCL (approx. 120 ng/cm^2^), Resomer® LT706 is covered with approx. 150 ng FN/cm^2^. However, the relation between absorbed FN and VN is the same for TCPS and Resomer® LT706 (fibronectin to vitronectin ratio = 1.2), while the relation between FN and VN on PCL is 1.04. These results were not significantly different either when we used a buffer system including FN and VN or serum-containing medium including FN and VN (not shown). Thus, the ratio of adsorbed FN to adsorbed VN might be key to MSC gene expression.

Finally, differentially regulated genes of MSC cultured on Resomer® LT706, PCl and TCPS under differentiation conditions (OIM) were subjected to hierarchical cluster analysis and data depicted in heat map format (Fig. 4B). This analysis shows that Resomer® LT706 and TCPS clustered together and PCL formed a distinct cluster. Some donor variations were also seen in this heat map representation, which is in line with the PCA analysis (Fig. 3A). In addition, this analysis identified a large cluster of genes, with a similar expression pattern in Resomer® LT706 and TCPS (Fig. 4B, upper boxed area). Furthermore, we also identified genes that showed a similar expression in MSC on Resomer® LT706 and PCL, but are differentially expressed in MSC on TCPS, e.g. CCL2 (Fig. 4B, bottom boxed area).

Well-known osteogenic marker genes were expressed in MSC cultured on all three biomaterials (TCPS, Resomer® LT706 and PCL; compare Fig. 2). Thus, the expression and interaction of other genes involved in osteogenesis and chondrogenesis (Fig. 4C) might be important to regulate MSC differentiation towards osteoblasts either via osteogenesis or ossification on these biomaterials. To further support the data of whole genome gene expression analysis, we perfomed quantitative PCR. Therefore, we choosed a panel of genes involved in skeletal development and osteogenic differentiation, highlighted in orange in Figure 4B. As shown in Figure 4C, gene expression of MSC-derived osteoblasts was influenced by biomaterial substrates or – by keeping in mind the results on FN and VN described above - by serum proteins which are adsorbed to the biomaterials before cell attachment.

Secreted frizzled related protein 4 (SFRP4), Proline/arginine-rich end leucine-rich repeat protein (PRELP), cartilage oligomeric matrix protein (COMP), α1 XI collagen (COL11A1) and elastin (ELN) expression is higher in MSC cultured in OIM on Resomer® LT706 and TCPS in comparison to PCL (Fig. 4C). Chemokine (C-C motif) ligand 2 (CCL2) expression is increased under differentiation conditions on Resomer® LT706 and PCL (compare Fig. 4C with Fig. 4B).

### Gene ontology (GO) overrepresentation analysis detects biological categories related to skeletal development and bone formation

Gene functions are described in a controlled vocabulary format referred to as gene ontology (GO; www.geneontology.org) and thus differentially regulated genes between the three biomaterials cultured in GM and OIM were subjected to GO overrepresentation analysis, respectively. The main categories of the two culture conditions are shown for MSC cultured on Resomer® LT706, PCL and TCPS in GM (Table 2) and for MSC cultured on Resomer® LT706, PCL and TCPS in OIM (Table S2). Some categories exist in both lists, like developmental process, system development and extracellular region. However, bone specific categories, such as bone remodelling, calcium ion binding, bone mineralisation, ossification and biomineral formation are – as expected - not found in the GO list for the growth conditions (GM), but confined to the GO list for the differentiation conditions (OIM).

**Table 2:** GO list with genes differentially expressed in MSC cultured on TCPS, Resomer® LT706 and PCL during growth conditions. (GM)

Term	Count	P-Value
**Biological Process**		
GO:0006817∼phosphate transport	5	114E-06
GO:0007275∼multicellular organismal development	18	237E-06
**GO:0032502∼developmental process**	21	288E-06
GO:0048856∼anatomical structure development	16	859E-06
GO:0015698∼inorganic anion transport	5	148E-05
GO:0006820∼anion transport	5	275E-05
GO:0048513∼organ development	12	310E-05
GO:0006029∼proteoglycan metabolic process	3	332E-05
GO:0022610∼biological adhesion	8	437E-05
GO:0007155∼cell adhesion	8	437E-05
GO:0048731∼system development	13	541E-05
GO:0016055∼Wnt receptor signaling pathway	4	620E-05
GO:0051216∼cartilage development	3	952E-05
GO:0032501∼multicellular organismal process	21	155E-04
GO:0030217∼T cell differentiation	3	190E-04
GO:0001501∼skeletal development	4	196E-04
GO:0046457∼prostanoid biosynthetic process	2	273E-04
GO:0001516∼prostaglandin biosynthetic process	2	273E-04
GO:0001502∼cartilage condensation	2	303E-04
GO:0006811∼ion transport	7	318E-04
GO:0006954∼inflammatory response	4	324E-04
GO:0002250∼adaptive immune response	3	364E-04
GO:0002460∼adaptive immune response based on somatic recombination of immune receptors	3	364E-04
GO:0006693∼prostaglandin metabolic process	2	422E-04
GO:0006692∼prostanoid metabolic process	2	422E-04
GO:0030098∼lymphocyte differentiation	3	427E-04
GO:0030199∼collagen fibril organization	2	451E-04
GO:0016525∼negative regulation of angiogenesis	2	568E-04
GO:0042110∼T cell activation	3	671E-04
GO:0009611∼response to wounding	4	738E-04
GO:0002521∼leukocyte differentiation	3	757E-04
GO:0046456∼icosanoid biosynthetic process	2	798E-04
GO:0006955∼immune response	5	894E-04
GO:0006690∼icosanoid metabolic process	2	966E-04
**Molecular Function**		
GO:0005201∼extracellular matrix structural constituent	7	312E-10
GO:0030020∼extracellular matrix structural constituent conferring tensile strength	5	159E-08
GO:0005515∼protein binding	29	150E-05
GO:0005198∼structural molecule activity	7	115E-04
GO:0005539∼glycosaminoglycan binding	3	245E-04
GO:0005488∼binding	42	280E-04
GO:0030247∼polysaccharide binding	3	291E-04
GO:0005125∼cytokine activity	4	317E-04
GO:0001871∼pattern binding	3	341E-04
GO:0005102∼receptor binding	6	509E-04
GO:0005506∼iron ion binding	4	774E-04
GO:0008083∼growth factor activity	3	828E-04
**Cellular Component**		
**GO:0005576∼extracellular region**	31	317E-16
GO:0044421∼extracellular region part	30	386E-16
GO:0005578∼proteinaceous extracellular matrix	13	405E-13
GO:0005615∼extracellular space	26	531E-13
GO:0031012∼extracellular matrix	13	553E-13
GO:0044420∼extracellular matrix part	7	532E-09
GO:0005581∼collagen	5	466E-08
GO:0005604∼basement membrane	4	103E-05
GO:0030935∼sheet-forming collagen	2	206E-04
GO:0005587∼collagen type IV	2	206E-04

The ‘Count’ column refers to the number of transcripts in the respective catergory.

The ‘P-Value’ column shows the value of Fisher's exact t-test, used by DAVID to measure the enrichment in annotation terms.

## Discussion

MSC are the natural precursor cells of mesenchymal tissue, such as fat, bone and cartilage. They reside in a tissue-specific niche, awaiting signals for tissue regeneration, if necessary [30]. In the last decade, MSC were extensively investigated for their usefulness in bone tissue engineering and diverse scaffold materials were suggested to provide an appropriate three-dimensional environment [31]–[34]. Promising tissue-engineered constructs are already on the way from bench to bedside [35]. However, the impact of scaffolds on osteogenic differentiation on the molecular level was rarely analysed and restricted to some well-known osteogenic marker genes, such as alkaline phosphatase, runt related transcription factor 2, type 1 collagen, bone sialoprotein and osteocalcin [34], [36].

In the present study, we initially investigated a panel of different polymers for their influence on the osteogenic differentiation of MSC. We then focused on the two most prominent synthetic polymers Resomer® LT706 and PCL. Both polymers are linear, semi-crystalline polyesters, which are hydrolytically or enzymatically degradable. Resomer® LT706 is degradable within 8–12 month, while PCL is long-term degradable (>24 month) [20].

PCL is an FDA proved and widely used engineering polymer with excellent physical, chemical and mechanical properties [37], [38]. It is already analysed in the context of bone tissue engineering, either as pure PCL [39], as composite scaffold e.g. together with hydroxyapatite or calcium phosphate [40], [41] or modified with a peptide layer such as RGD [16]. All of these studies suggest PCL as suitable scaffold material for bone tissue engineering.

In contrast, Resomer® LT706 is a relatively novel and less-investigated synthetic polymer, described for the first time as Poly(L-lactide-co-TMC) in 2005 by Pospiech and coworkers [42]. Materials with related chemistry are shown to be biodegradable and biocompatible for soft tissue engineering [19] and used in clinical trials for skin replacements [17], [18]. However, our recent work showed that a prediction of cell behaviour on a chemically related material is not possible [5]. Resomer® LT706 was not analysed in the context of bone tissue engineering or osteogenic differentiation of MSC so far.

We compared the osteogenic differentiation of MSC on the two elastomeric and long-term degradable synthetic polymers Resomer® LT706 and PCL. TCPS served as control. Our XPS data revealed that the two polyesters Resomer® LT706 and PCL do not differ qualitatively in their elemental composition, but quantitatively, indicated by the C/O ratio. The C/O-values for Resomer® LT706 and PCL are 1.7 and 2.6, respectively (Table S1). Further, XPS analysis of our biomaterial surfaces shows that PCL contains less oxygen atoms than Resomer® LT706, indicating PCL as more hydrophobic than Resomer® LT706. However, contact angle measurements detected the opposite, with Resomer® LT706 being more hydrophobic than PCL with the corresponding contact angles of 75° and 69°, respectively. The higher hydrophobic characteristic of Resomer® LT706 is a result of its molecular structure. Resomer® LT706 consists of 68–72% L-lactide units and thus includes sterically demanding and hydrophobic methyl groups in high frequencies at the surface. In contrast, PCL only includes methylene groups in its backbone. TCPS – in contrast to polystyrol – does not consist of phenyl rings, but also includes surface modifications (established by physical plasma) of hydroxyl-, carboxyl- and amino-groups on the surface and a contact angle of only 54° (Table S1).

To allow for an adequate adhesion of MSC on our substrates (TCPS, Resomer® LT706, PCL and TCPS), an initial adsorption of cell adhesion mediators from culture medium, such as fibronectin (FN) and vitronectin (VN), is crucial, since in general, the less adsorbed proteins the less cell adhesion is mediated. As shown in our adsorption studies with radiolabelled FN and VN (Figure S1), Resomer® LT706 is covered with the highest amount of FN proteins, compared to TCPS and PCL. Further, the ratio of FN to VN on Resomer® LT706 is comparable to that on TCPS, albeit absolut values are higher for Resomer® LT706. It is known that extracellular matrix (ECM) proteins are involved in cell adhesion not only by their own presence, but also by their relation to other ECM proteins [43], [44]. This ratio of different ECM proteins then can result in differences in cell adhesion, morphology, proliferation, gene expression as well as ECM secretion. The similar ratio of FN/VN on Resomer® LT706 and TCPS compared to PCL (1.2 and 1.04, respectively) might be the reason for a comparable initial adhesion and morphology of MSC, which differs from that on PCL, where the cells are more roundish after 24 h of incubation [5]. However, MSC proliferation within 7 days after cell seeding was comparable for TCPS, Resomer® LT706 and PCL [5] and cell morphology was identical after 21 days of culture in GM or OIM (Fig. 1C). Although we detected a comparable proliferation of MSC on all three substrates, osteogenic differentiation capacity was different.

In the present study, we found that both Resomer® LT706 and PCL cyused stronger alizarin red stainings compared to TCPS, which however was most significant for Resomer® LT706. The fact that the alizarin red staining is stronger for Resomer® LT706 than for PCL, though MSC proliferation is comparable on both polymers [5], suggests that Resomer® LT706 is more potent in supporting osteogenic differentiation than PCL. A positive Alizarin red staining is indicative for osteogenic MSC fate, but stains only calcium accumulations and thus provides only indirect evidence. We were thus interested in the quality and the molecular mechanisms underlying osteogenic differentiation of MSC on Resomer® LT706 and PCL. As initial step in this direction we performed whole genome expression analysis using Affymetrix gene arrays. Bioinformatic tools then unravelled (i) donor variations as expected for primary cells, (ii) clusters of samples representative for MSC cultured in growth medium (GM) or in osteogenic induction medium (OIM), indicating a stronger influence of culture medium than of biomaterial substrates, and (iii) clusters of TCPS and Resomer® LT706 under growth (GM) and differentiation conditions (OIM), demonstrating a similar influence on MSC gene expression. Pairs of TCPS and Resomer® LT706 were detected in all bioinformatic analysis. Although both materials are quite different e.g. in surface chemistry (polarity, hydrophobicity, surface charge) and bulk properties (e.g. stiffness), the impact on gene expression related to osteogenesis of MSC is comparable. Yet, TCPS is not useful for bone tissue engineering because of its fabrication characteristics based on its chemical and physical properties. Again this supports our recent study, showing that related biomaterials do not inevitably result in the same cellular response, while unrelated materials might do [5]. The heat map in Figure 4B shows that most genes were expressed on a similar level in MSC cultured on TCPS and Resomer® LT706, while only a few genes were differentially expressed on these two materials.

We extended our gene array results by RT-qPCR of the six genes secreted frizzled related protein 4 (SFRP4), Proline/arginine-rich end leucine-rich repeat protein (PRELP), cartilage oligomeric matrix protein (COMP), α1 XI collagen (COL11A1), elastin (ELN) and chemokine (C-C motif) ligand 2 (CCL2) (Fig. 4C).

For all of these genes published data suggest a role in skeletal development or osteogenic differentiation. SFRP4 is expressed in periosteum and bone tissue but overexpression of SFRP4 suppresses osteoblast proliferation [45]. PRELP is a connective tissue matrix protein, which is expressed in cartilage and in osteoblasts [46]. COMP is involved in skeletal development and osteoblast differentiation [47] and detectable in MG-63 cells, an osteoblast cell line. Mutations of COMP are related to specific diseases, such as pseudoachondroplasia and multiple epiphyseal dysplasia [48]. COL11A1 is essential for normal skeletal development, but has to be suppressed for terminal osteoblast differentiation [49]. Elastin degradation products promote osteogenic differentiation and elastin-related calcification is suggested to be involved in tissue repair processes [50]. Finally, CCL2 - which is known as chemokine for the recruitment of cells of the immune system, such as monocytes - is secreted by MSC and this secretion increases the differentiation into mature osteoblasts [51].

Compared to MSC cultured on TCPS and Resomer® LT706, all genes showed lower expression in MSC cultured on PCL, except CCL2. Since all these genes are involved in skeletal development and bone formation, our results suggest Resomer® LT706 as more suitable for bone tissue engineering than PCL.

In summary, we analysed osteogenic differentiation of MSC on Resomer® LT706 and PCL. Alizarin red stainings and expression of conventional osteogenic transcripts show that both biomaterials support osteogenic differentiation fate. However, whole genome expression analysis revealed differences in gene expression and genes involved in skeletal development and bone formation are more expressed at higher levels in MSC cultured on Resomer® LT706. Thus, this novel, long-term degradable and osteoconductive synthetic polymer is suggested as particularly attractive scaffold material for bone tissue engineering with superior properties compared to the currently being used material PCL. The *in vitro* transformation of MSC on Resomer® LT706 to more osteogenic genotypes might also translate to phenotypes. This hypothesis has to be verified in future *in vivo* models.

## Supporting Information

Figure S1 **Adsorption of serum proteins fibronectin (FN) and vitronectin (VN) on biomaterial surfaces.** TCPS, Resomer® LT706 and PCL are coated with radiolabelled FN and VN. Adsorbed proteins are quantified using a Gammacounter COBRA II device (ng adsorbed protein per cm^2^ biomaterial surface). Mean values of 10 independent measurements per coating are shown; *p<0.01 compared to TCPS.
(JPG)

Figure S2 **Characterisation of MSC according to minimal criteria of the International Society for Cellular Therapy.** MSC can be differentiated according to standard protocols towards adipocytes, osteoblasts and chondrocytes (A) and express a specific surface pattern with positivity for CD 73, CD90 and CD105 and without expression of hematopoietic markers, such as CD4 and CD14.
(JPG)

Figure S3 **Control staining of polymers without stem cells after incubation in OIM.** The polymers do not bind Alizarin red stain after 21 days of incubation in OIM.
(JPG)

Table S1 **Characterisation of biomaterials.**
(DOC)

Table S2 **GO list with genes differentially expressed in MSC cultured on TCPS, Resomer® LT706 and PCL during differentiation conditions (OIM).**
(DOC)
