# Supplementary figures and images for: Transcriptome Analysis of MSC and MSC-Derived Osteoblasts on Resomer® LT706 and PCL: Impact of Biomaterial Substrate on Osteogenic Differentiation
Source: PLoS One. 2011 Sep 14;6(9):e23195. doi: 10.1371/journal.pone.0023195 (PMC3173366; doi:10.1371/journal.pone.0023195)

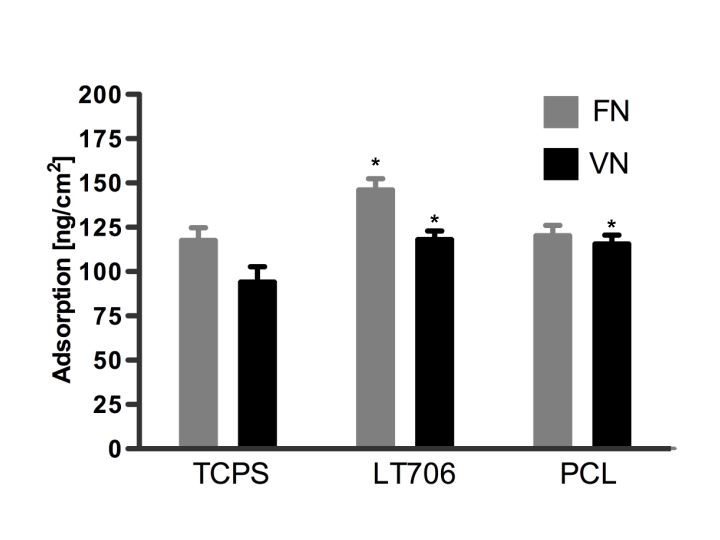

Supplement: Figure S1 — Adsorption of serum proteins fibronectin (FN) and vitronectin (VN) on biomaterial surfaces. TCPS, Resomer® LT706 and PCL are coated with radiolabelled FN and VN. Adsorbed proteins are quantified using a Gammacounter COBRA II device (ng adsorbed protein per cm2 biomaterial surface). Mean values of 10 independent measurements per coating are shown; *p<0.01 compared to TCPS. (JPG) [file pone.0023195.s001.jpg]

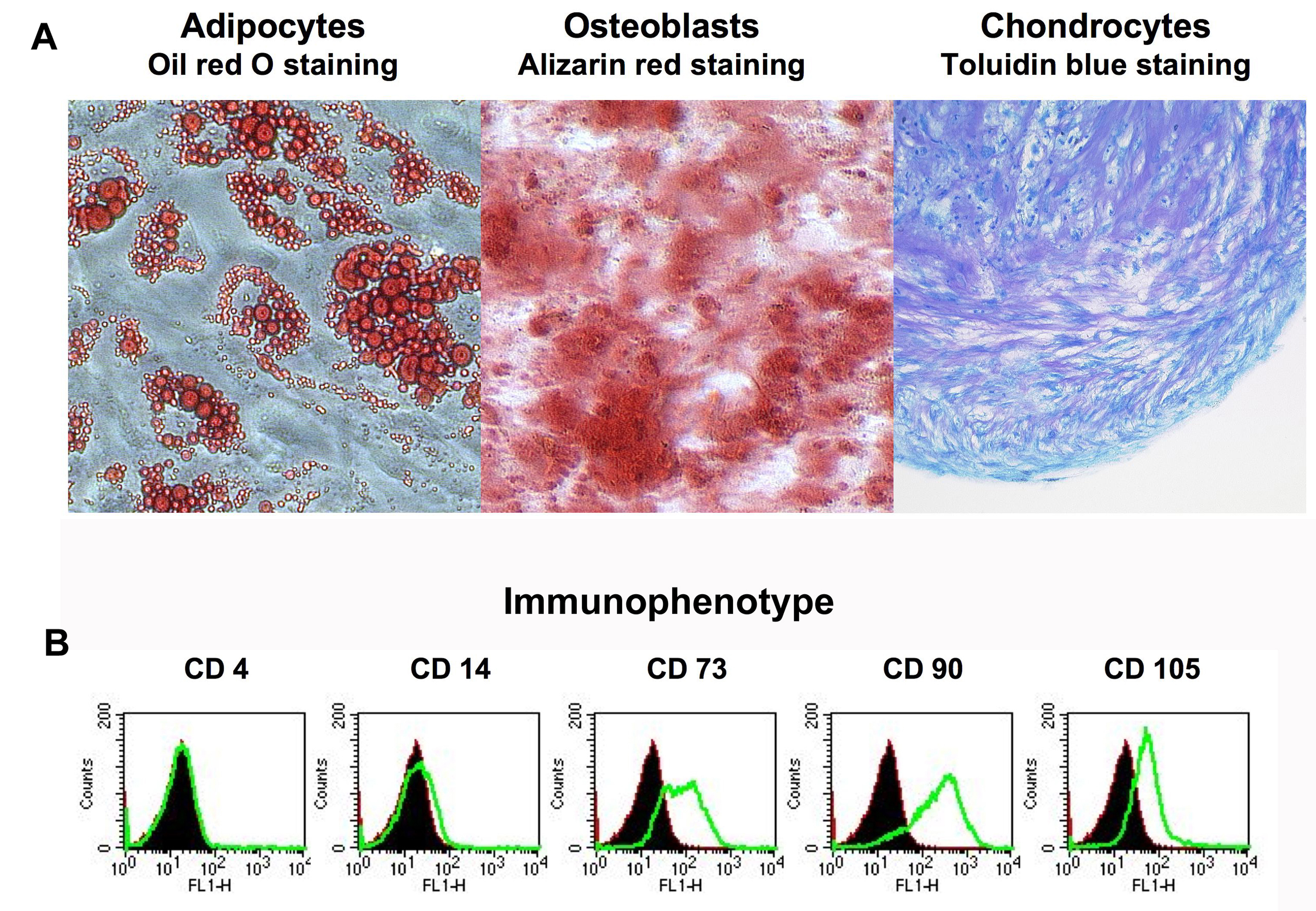

Supplement: Figure S2 — Characterisation of MSC according to minimal criteria of the International Society for Cellular Therapy. MSC can be differentiated according to standard protocols towards adipocytes, osteoblasts and chondrocytes (A) and express a specific surface pattern with positivity for CD 73, CD90 and CD105 and without expression of hematopoietic markers, such as CD4 and CD14. (JPG) [file pone.0023195.s002.jpg]

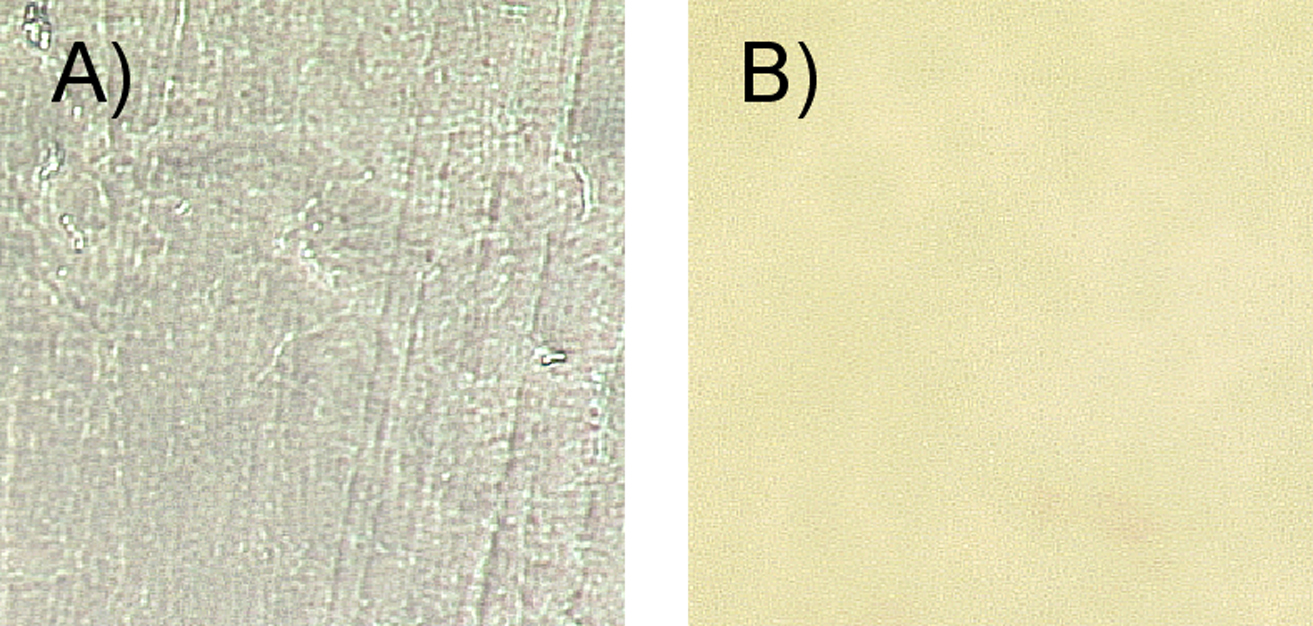

Supplement: Figure S3 — Control staining of polymers without stem cells after incubation in OIM. The polymers do not bind Alizarin red stain after 21 days of incubation in OIM. (JPG) [file pone.0023195.s003.jpg]
