# Supplementary material for: Transcriptome Analysis of MSC and MSC-Derived Osteoblasts on Resomer® LT706 and PCL: Impact of Biomaterial Substrate on Osteogenic Differentiation
Source: PLoS One. 2011 Sep 14;6(9):e23195. doi: 10.1371/journal.pone.0023195 (PMC3173366; doi:10.1371/journal.pone.0023195)
Supplement: Table S1 — Characterisation of biomaterials. (DOC) [file pone.0023195.s004.doc]

| Material | Roughness R_a_  [nm] | Elemental composition  [atom-%]  C O N others | Contact Angle  [°] |
| --- | --- | --- | --- |
| TCPS | 10 ± 1 | 80.4 18.7 0.9 -- | 54 ± 2 |
| PCL | 40 ± 3 | 72.4 27.6 -- -- | 69 ± 2 |
| P(LLA-co-TMC)_7/3_ | 41 ± 2 | 63.0 37.0 -- -- | 75 ± 2 |
