# Supplementary material for: Transcriptome Analysis of MSC and MSC-Derived Osteoblasts on Resomer® LT706 and PCL: Impact of Biomaterial Substrate on Osteogenic Differentiation
Source: PLoS One. 2011 Sep 14;6(9):e23195. doi: 10.1371/journal.pone.0023195 (PMC3173366; doi:10.1371/journal.pone.0023195)
Supplement: Table S2 — GO list with genes differentially expressed in MSC cultured on TCPS, Resomer® LT706 and PCL during differentiation conditions (OIM). (DOC) [file pone.0023195.s005.doc]

Table S2

| **Term** | **Count** | **P-Value** |
| --- | --- | --- |
| Biological Process |  |  |
| GO:0050930~induction of positive chemotaxis | 4 | 259E-08 |
| GO:0050927~positive regulation of positive chemotaxis | 4 | 259E-08 |
| GO:0050926~regulation of positive chemotaxis | 4 | 259E-08 |
| GO:0050921~positive regulation of chemotaxis | 4 | 388E-08 |
| GO:0050920~regulation of chemotaxis | 4 | 388E-08 |
| GO:0050918~positive chemotaxis | 4 | 388E-08 |
| GO:0009605~response to external stimulus | 10 | 730E-07 |
| GO:0008283~cell proliferation | 9 | 724E-06 |
| GO:0006955~immune response | 9 | 876E-06 |
| GO:0009611~response to wounding | 7 | 106E-05 |
| GO:0048771~tissue remodeling | 5 | 107E-05 |
| GO:0007155~cell adhesion | 9 | 316E-05 |
| GO:0022610~biological adhesion | 9 | 316E-05 |
| GO:0002376~immune system process | 10 | 379E-05 |
| **GO:0032502~developmental process** | 21 | 400E-05 |
| **GO:0030282~bone mineralization** | 3 | 407E-05 |
| GO:0001501~skeletal development | 5 | 489E-05 |
| **GO:0001503~ossification** | 4 | 496E-05 |
| **GO:0031214~biomineral formation** | 4 | 511E-05 |
| GO:0045087~innate immune response | 4 | 589E-05 |
| GO:0007275~multicellular organismal development | 17 | 614E-05 |
| **GO:0046849~bone remodeling** | 4 | 657E-05 |
| GO:0006935~chemotaxis | 4 | 825E-05 |
| GO:0042330~taxis | 4 | 825E-05 |
| GO:0051179~localization | 20 | 904E-05 |
| GO:0006954~inflammatory response | 5 | 958E-05 |
| GO:0006950~response to stress | 9 | 110E-04 |
| GO:0051216~cartilage development | 3 | 134E-04 |
| GO:0006952~defense response | 8 | 166E-04 |
| GO:0006811~ion transport | 8 | 239E-04 |
| GO:0009888~tissue development | 5 | 247E-04 |
| GO:0051239~regulation of multicellular organismal process | 5 | 266E-04 |
| GO:0032501~multicellular organismal process | 23 | 326E-04 |
| GO:0048856~anatomical structure development | 14 | 327E-04 |
| GO:0048514~blood vessel morphogenesis | 4 | 343E-04 |
| GO:0007610~behavior | 5 | 353E-04 |
| GO:0006957~complement activation, alternative pathway | 2 | 361E-04 |
| GO:0008284~positive regulation of cell proliferation | 4 | 366E-04 |
| GO:0051145~smooth muscle cell differentiation | 2 | 397E-04 |
| GO:0006810~transport | 16 | 460E-04 |
| GO:0007626~locomotory behavior | 4 | 464E-04 |
| GO:0000074~regulation of progression through cell cycle | 5 | 470E-04 |
| GO:0006812~cation transport | 6 | 475E-04 |
| GO:0051726~regulation of cell cycle | 5 | 482E-04 |
| GO:0001568~blood vessel development | 4 | 496E-04 |
| GO:0030500~regulation of bone mineralization | 2 | 502E-04 |
| **GO:0048731~system development** | 12 | 507E-04 |
| GO:0001944~vasculature development | 4 | 512E-04 |
| GO:0065007~biological regulation | 24 | 538E-04 |
| GO:0051234~establishment of localization | 16 | 564E-04 |
| GO:0050778~positive regulation of immune response | 3 | 598E-04 |
| GO:0048523~negative regulation of cellular process | 8 | 613E-04 |
| GO:0051049~regulation of transport | 3 | 617E-04 |
| GO:0002684~positive regulation of immune system process | 3 | 617E-04 |
| GO:0030154~cell differentiation | 12 | 699E-04 |
| GO:0048869~cellular developmental process | 12 | 699E-04 |
| GO:0048513~organ development | 10 | 714E-04 |
| GO:0016044~membrane organization and biogenesis | 4 | 734E-04 |
| GO:0016055~Wnt receptor signaling pathway | 3 | 739E-04 |
| GO:0050776~regulation of immune response | 3 | 760E-04 |
| GO:0002682~regulation of immune system process | 3 | 782E-04 |
| GO:0048518~positive regulation of biological process | 8 | 796E-04 |
| GO:0030278~regulation of ossification | 2 | 812E-04 |
| GO:0030595~leukocyte chemotaxis | 2 | 812E-04 |
| GO:0048519~negative regulation of biological process | 8 | 812E-04 |
| GO:0030111~regulation of Wnt receptor signaling pathway | 2 | 846E-04 |
| GO:0051240~positive regulation of multicellular organismal process | 3 | 846E-04 |
| GO:0045807~positive regulation of endocytosis | 2 | 946E-04 |
| GO:0050793~regulation of developmental process | 4 | 977E-04 |
| GO:0046850~regulation of bone remodeling | 2 | 980E-04 |
| GO:0006029~proteoglycan metabolic process | 2 | 980E-04 |
| GO:0016477~cell migration | 4 | 992E-04 |
|  |  |  |
| **Molecular Function** |  |  |
| GO:0008083~growth factor activity | 8 | 290E-08 |
| GO:0005125~cytokine activity | 7 | 280E-06 |
| GO:0004866~endopeptidase inhibitor activity | 6 | 587E-06 |
| GO:0030414~protease inhibitor activity | 6 | 635E-06 |
| GO:0005102~receptor binding | 10 | 120E-05 |
| GO:0004857~enzyme inhibitor activity | 6 | 282E-05 |
| **GO:0005509~calcium ion binding** | 10 | 702E-05 |
| GO:0004867~serine-type endopeptidase inhibitor activity | 4 | 129E-04 |
| GO:0042056~chemoattractant activity | 2 | 155E-04 |
| GO:0004947~bradykinin receptor activity | 2 | 194E-04 |
| GO:0030234~enzyme regulator activity | 8 | 232E-04 |
| GO:0005539~glycosaminoglycan binding | 3 | 400E-04 |
| GO:0030247~polysaccharide binding | 3 | 474E-04 |
| GO:0001871~pattern binding | 3 | 552E-04 |
| GO:0005104~fibroblast growth factor receptor binding | 2 | 717E-04 |
| GO:0001653~peptide receptor activity | 3 | 801E-04 |
|  |  |  |
| **Cellular Component** |  |  |
| GO:0044421~extracellular region part | 36 | 159E-16 |
| GO:0005615~extracellular space | 35 | 188E-16 |
| **GO:0005576~extracellular region** | 37 | 246E-16 |
| GO:0005578~proteinaceous extracellular matrix | 8 | 181E-06 |
| GO:0031012~extracellular matrix | 8 | 213E-06 |
| GO:0044420~extracellular matrix part | 4 | 812E-05 |
| GO:0005604~basement membrane | 3 | 300E-04 |
|  |  |  |
|  |  |  |
| #The 'Count' column refers to the number of transcripts in the respective catergory. | | |
| #The 'P-Value' column shows the value of Fisher's exact t-test, used by DAVID to measure the enrichment in annotation terms | | |
